# Sleep State Misperception in Insomnia: The Role of Sleep Instability and Emotional Dysregulation

**DOI:** 10.3390/brainsci15101078

**Published:** 2025-10-04

**Authors:** Elettra Cini, Francesca Bolengo, Elisabetta Fasiello, Francesca Berra, Maurizio Gorgoni, Marco Sforza, Francesca Casoni, Paola Proserpio, Vincenza Castronovo, Luigi De Gennaro, Luigi Ferini-Strambi, Andrea Galbiati

**Affiliations:** 1 Faculty of Psychology, Vita-Salute San Raffaele University, 20132 Milan, Italy; elettra.cini@gmail.com (E.C.); francibolengo@gmail.com (F.B.); francescaberra1997@gmail.com (F.B.);; 2 Department of Clinical Neurosciences, Neurology-Sleep Disorders Center, IRCCS San Raffaele Scientific Institute, 20132 Milan, Italy; marco.sforza@gmail.com (M.S.); casoni.francesca@hsr.it (F.C.);; 3 IUSS Cognitive Neuroscience (ICON) Center, Scuola Universitaria Superiore IUSS, 27100 Pavia, Italy; 4 Department of Psychology, Sapienza University of Rome, 00185 Rome, Italy; maurizio.gorgoni@uniroma1.it (M.G.);; 5 Body and Action Lab, IRCCS Fondazione Santa Lucia, 00179 Rome, Italy

**Keywords:** insomnia disorder, sleep state misperception, sleep instability, polysomnography, emotional dysregulation, hyperarousal theory, cortical arousals

## Abstract

**Background/Objectives:** Sleep state misperception (SSM) is a common phenomenon in insomnia disorder (ID), characterized by a discrepancy between subjective and objective sleep metrics. Recent studies have revealed microstructural EEG alterations specifically in misperceiving ID patients, yet clinically accessible SSM markers remain limited. This study aimed to characterize SSM within ID by integrating standard polysomnography (PSG) features and cognitive-affective traits, focusing on accessible clinical tools. **Methods:** Twenty patients with ID and twenty healthy controls (HC) underwent a night of PSG recording and completed both sleep diaries and a comprehensive psychological assessment. SSM was quantified using the Total Sleep Time misperception index (TSTm), analyzed both dimensionally and categorically **Results:** IDs reported significantly altered sleep parameters compared to HCs, both subjectively and objectively. Within the ID sample, although underestimators and normoestimators had similar objective TST, underestimators showed significantly more cortical arousal density (CAd), a higher percentage of sleep stage 1 and higher non-acceptance of emotions. Notably, none of the HC reached the threshold for being classified as underestimators. Regression analyses identified CAd, latency to sleep stage 3 and to REM, percentage of REM sleep and lack of emotional clarity, as key predictors of TSTm. **Conclusions:** SSM in insomnia reflects a dimensional vulnerability grounded in subtle sleep fragmentation and emotional dysregulation. Recognizing SSM as a clinically meaningful phenomenon may guide more targeted, emotion-focused, interventions for insomnia.

## 1. Introduction

Insomnia disorder (ID) is the most prevalent sleep disorder and the second most common mental disorder globally [1]. Defined by persistent difficulties in sleep initiation, maintenance, or early morning awakenings despite adequate opportunity to sleep, ID is diagnosed solely based on subjective complaints [2]. This reliance on subjective experience is not a flaw but reflects the phenomenological essence of insomnia.

A particularly striking behavioral expression of this subjectivity is the discrepancy between perceived and objectively recorded sleep, known as sleep state misperception (SSM) [3], or paradoxical insomnia [4]. Individuals with SSM may subjectively report sleeping very little, or not at all, even though polysomnographic (PSG) recordings show normal or near-normal sleep durations [5]. Far from anecdotal, this paradox underscores a fundamental dissociation between sleep macrostructure and subjective experience, raising questions about how the brain constructs the subjective sense of sleep and whether this experience reflects transient states or stable individual characteristics [6,7].

Standard PSG metrics, such as total sleep time (TST), sleep efficiency (SE), or sleep stage distribution, have shown limited explanatory power in accounting for this mismatch [8,9]. Indeed, many individuals with ID show preserved macrostructural sleep despite severe complaints [10]. Although the phenomenon of SSM has long attracted clinical and scientific interest, it has traditionally been framed as a perceptual distortion on the part of the patient. This reductive interpretation contributed to the marginalization of SSM as a distinct clinical phenotype. As highlighted by Stephan [11], research initially focused on macrostructural sleep parameters, which have proven insufficient to explain the subjective-objective mismatch. More recent work has shifted toward microstructural and EEG-based approaches, offering new insights into the underlying mechanisms of SSM. These investigations have revealed that patients with ID, especially those with misperception, exhibit persistent cortical hyperactivation during sleep, reflected in reduced delta power, increased beta and sigma activity, and flattened 1/f slopes [8,11,12]. Among these markers, a persistently low delta/beta ratio, an index of cortical hyperarousal, has been proposed as a neurophysiological correlate of SSM [7]. These insights have prompted a conceptual shift: rather than attributing SSM to cognitive distortions in the patient’s subjective evaluation of sleep, some authors have suggested that it may instead reflect the inability of conventional PSG to detect relevant neurophysiological features underlying sleep perception, reframing the issue as one of mismeasurement rather than misperception [11]. However, such quantitative EEG indices require advanced analytic techniques, limiting their clinical applicability.

More accessible PSG-based features, such as cortical arousals, may offer clinically viable insights. These brief, sub-threshold EEG activations can fragment sleep continuity without reaching full wakefulness and may significantly contribute to the subjective perception of disturbed sleep [9,13]. Notably, increased rapid eye movements (REM) sleep arousal density has been associated with both emotional dysregulation and a shift in sleep mentation toward more cognitive, thought-like content [14], suggesting a potential link between sleep fragmentation and affective instability in shaping sleep misperception.

Building on this interplay between sleep physiology and subjective experience, cognitive and emotional factors have also been increasingly recognized as playing a key role in shaping sleep perception. In addition to physiological markers, cognitive-affective traits, such as emotion dysregulation (e.g., difficulty accepting emotional states) and maladaptive metacognitive beliefs (e.g., worry and rumination about sleep), have been proposed as psychological mechanisms that intensify attention to perceived sleep disturbances [15,16]. These traits are conceptually embedded within the broader hyperarousal framework, which spans physiological, cognitive, and emotional dimensions [17]. Nonetheless, it is not yet known whether these traits underlie a stable misperception phenotype or interact dynamically with nightly sleep processes.

Although growing evidence supports the involvement of specific neurophysiological and psychological mechanisms in SSM, the extent to which these reflect stable clinical traits or state-dependent vulnerabilities remains unclear. Notably, SSM does not consistently co-occur with general psychopathology, as confirmed by Castelnovo et al. (2021), who found no significant differences in psychopathological profiles between ID patients with high and moderate underestimators [10].

Furthermore, the relationship between SSM and perceived insomnia severity remains unclear. While SSM and ID often co-occur, it is still debated whether higher degrees of misperception correspond to disorder and symptoms severity [10,18,19,20,21]. Clarifying this relationship is essential to determine whether SSM constitutes a marker of ID severity, a distinct vulnerability profile, or a dynamic interaction between physiological instability and emotional processing.

The present study aims to answer these open questions by providing a multidimensional characterization of SSM. To this end, we integrated clinically accessible PSG parameters, particularly cortical arousals, with validated psychological measures assessing emotion regulation (Difficulties in Emotion Regulation Scale, DERS) [22], metacognitive beliefs (Metacognitions Questionnaire–Insomnia, MCQ-I) [23], and insomnia severity (Insomnia Severity Index, ISI) [24], in a sample comprising healthy controls (HC) and individuals with ID. Total Sleep Time misperception index (TSTm), calculated as the ratio between subjective and objective sleep [25], was analyzed both as a continuous variable (capturing dimensional variations in the subjective-objective alignment of sleep perception) and categorically (distinguishing underestimators from normoestimators based on the validated cut-off value [9]).

By combining accessible PSG and psychological measures, this study aims to identify vulnerability factors associated with SSM, contributing to a dimensional understanding of sleep misperception as a potentially dynamic and affectively mediated phenomenon within insomnia. Gaining insight into these mechanisms may support more precise case conceptualization, and help integrate the patient’s subjective experience, particularly its emotional dimension, more effectively into clinical assessment and treatment planning.

## 2. Materials and Methods

### 2.1. Participants and Experimental Procedures

This study includes a sample of twenty-two patients diagnosed with ID (mean age: 43.50 ± 12.75; 7 M/13 F), recruited among the patients of the Sleep Disorder Centre of San Raffaele Hospital (Milan, Italy), and twenty HCs (mean age: 41.05 ± 11.55; 10 M/10 F). Two ID participants were excluded from the analyses after a visual inspection of the PSG recordings due to technical issues. Consequently, the final analysis was conducted on 20 ID patients and 20 HC participants. Macrostructural (i.e., standard PSG measures) and quantitative EEG (i.e., power EEG analysis) from the same sample have been presented in a previous publication [7] which addressed different research questions.

The recruitment of ID patients involved an initial evaluation by a neurologist specializing in sleep medicine. All patients met the diagnostic criteria for ID as defined by the International Classification of Sleep Disorders—Third Edition (ICSD-3) [2]. Exclusion criteria included: (i) presence of dementia, psychiatric conditions, or other sleep disorders; (ii) neurological comorbidities; and (iii) use of medications or substances affecting sleep, mood, or central nervous system activity. Patients were not under treatment with benzodiazepines, antidepressants, or any other psychoactive medications at the time of the PSG recording.

HC participants were recruited from the general population and selected based on the following cut-off scores: the Insomnia Severity Index (ISI) [24] score of <8 [26], the Epworth Sleepiness Scale (ESS) [27] score of <10 [28] and the Pittsburgh Sleep Quality Index (PSQI) [29] score of <5 [30], in order to, respectively, exclude individuals with insomnia, excessive daytime sleepiness, or other sleep-related issues.

HCs were instructed to maintain a regular sleep–wake schedule for the week preceding PSG assessment, verified through daily sleep diaries completed each morning within 15 min of awakening. Additionally, HCs were required to be free of any medications or substances that could interfere with sleep, mood, or central nervous system functioning for at least two months prior to the experimental session.

In addition to the questionnaires previously mentioned, both HC and ID participants completed a comprehensive battery of psychological assessments presented in Table 1.

#### Missing Data

Several participants in the ID sample had missing data for certain questionnaires, which were accounted for during the analysis. Specifically: (i) three patients did not have any available test data; (ii) two patients were missing data for the DERS; (iii) one participant was missing data for MCQI.

These missing values were addressed by excluding participants from specific analyses where the relevant data was unavailable.

### 2.2. Sleep Recording

On the day of the experimental session, each participant arrived at the Sleep Disorder Center of San Raffaele Hospital (Milan) between 5:30 and 7:00 p.m. to undergo an ambulatory PSG recording. Sleep monitoring was performed in participants’ private homes to promote ecological validity and minimize the first-night effect. Participants were instructed to abstain from consuming caffeine and alcohol during the afternoon preceding the recording. Lights-off time was aligned with individual’s habitual bedtime, and participants were allowed to wake spontaneously in the morning.

PSG recordings were conducted using 19 cortical EEG channels arranged according to the international 10–20 system [37]. Signals were acquired through unipolar derivations referenced to the averaged mastoids (A1–A2), with filtering parameters set to include all conventional EEG frequency bands: a high-pass filter at 0.5 Hz and an antialiasing low-pass filter at 35 Hz. Electrooculography (EOG) and electromyography (EMG) from the submentalis muscle were also recorded. Impedance for EEG channels was maintained below 5 kΩ throughout the recordings.

Sleep stages and artifact rejection were manually scored by an experienced sleep researcher, following the American Academy of Sleep Medicine (AASM) guidelines [38], based on 30 s epochs. Epochs containing ocular or muscular artifacts were manually excluded. Scoring N3 strictly adhered to the amplitude criterion of >75 μV.

Cortical arousals were identified based on abrupt shifts in EEG frequency, including theta, alpha, or frequencies exceeding 16 Hz (excluding sleep spindles), lasting at least 3 s and no more than 15 s, preceded by a minimum of 10 s of stable sleep. While objective awakenings were operationalized as EEG-defined wake periods lasting ≥ 60 s. [38].

The macrostructural sleep parameters extracted are presented in Table 2.

### 2.3. Sleep Diaries

To verify adherence to regular sleep–wake patterns, all HCs completed a seven-day sleep diary during the week preceding the PSG assessment. Additionally, both HC and ID participants were instructed to complete a sleep diary in the morning following the PSG recording. The diary was used to collect subjective sleep data referring to each previous night’s sleep. Diaries were to be filled out within 15 min of final awakening to reduce potential recall bias and capture immediate post-sleep subjective experiences and perceptions, including perceived sleep quality.

The subjective sleep parameters extracted from the diaries are presented in Table 3.

### 2.4. Sleep State Misperception Index: TSTm

TSTm, defined as the discrepancy between subjective and objective estimation of sleep duration over the entire night [25], was calculated by comparing subjective and objective indices derived from the sleep diary and PSG recordings, respectively, collected on the experimental night. The TSTm index, representing the extent of SSM across the full night, was computed using the following formula: TSTm = (sTST/oTST) × 100. A TSTm value of 100% indicated full concordance between subjective and objective sleep estimates (i.e., accurate perception of TST). Values lower than 100% reflected an underestimation of oTST, while values greater than 100% represented overestimation.

The TSTm index was considered as a continuous variable for correlational analyses.

However, to examine group differences and characterize participants based on the degree of SSM, a categorical classification was applied using validated cut-off values [9]. While none of the HCs met the criteria for misperception, ID participants were subdivided into two subgroups: (i) ID + TSTm (underestimators), comprising patients who significantly underestimated their oTST (TSTm < 88.31%); (ii) ID–TSTm (normoestimators), comprising patients whose TSTm values fell within the normative estimation range (88.31% ≤ TSTm ≤ 110.43%). This stratification enabled analyses aimed at comparing clinical, cognitive, and physiological variables between misperceiving and non-misperceiving insomnia patients.

### 2.5. Statistical Analysis

Statistical analyses were performed using JASP software (version 0.11.1 & version 0.19.3; JASP Team, Amsterdam, The Netherlands) and RStudio (version 2021.09.0; RStudio, PBC, Boston, MA, USA). Since the sample size is relatively small, non-parametric tests were used. The statistical analysis was conducted first between the ID and HC groups, and then between the ID + TSTm group (ID patients who underestimated their oTST) and the ID-TSTm group (ID patients who did not misperceive their oTST), according to the previously defined cut-off values [9]. Data are presented as means ± standard deviations (SD). For HC versus ID comparisons, HC values are reported first; similarly, in ID-TSTm versus ID + TSTm comparisons, values for ID-TSTm precede those for ID + TSTm.

To investigate the relationships between variables, Spearman rho’s correlations were calculated for both the ID and HC groups, as well as for the ID + TSTm and ID-TSTm subgroups. They are reported in Supplementary Results for descriptive and exploratory purposes. Regression analyses, performed using a stepwise approach, are presented as the primary method to test directional and potentially causal relationships, identifying predictors of sleep misperception and other relevant outcomes.

All statistical tests were conducted with a significance level of *p* < 0.05. Given the exploratory nature of the study, *p*-values are reported without correction for multiple comparisons [39,40].

## 3. Results

### 3.1. HCs vs. IDs

#### 3.1.1. Clinical and Psychological Variables

Table 4 provides an overview of demographic characteristics and questionnaire outcomes. Without considering the SSM phenomenon, IDs significantly differed from HCs on several psychological measures.

#### 3.1.2. Subjective and Objective Sleep Variables

**Figure 1:**
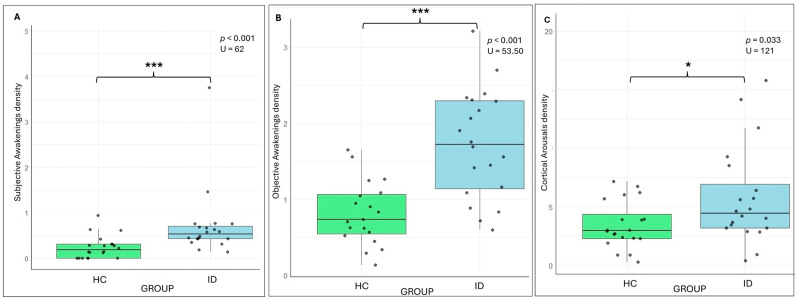
Boxplots illustrating subjective (self-reported; **A**) and objective (PSG-derived; **B**) nocturnal awakenings density and cortical arousal density (**C**) in HC (in light blue) and ID patients (in light green). Abbreviations: Healthy Controls (HC); Insomnia Disorder (ID); subjective number of awakenings density (sAWKd); objective number of awakenings density (oAWKd); number of cortical arousal density (CAd). *p* < 0.05 *, *p* < 0.001 ***.

During the experimental night, IDs reported significantly altered sleep parameters compared to HCs, both subjectively and objectively. In particular, they described a higher density of nocturnal awakenings in their sleep diaries, calculated as the number of awakenings per hour of sleep (U = 62, *p* < 0.001; HC: 0.24 ± 0.25 ID: vs. 0.72 ± 0.77). This pattern was mirrored by PSG-derived data, which confirmed a significantly higher objective nocturnal awakening density (U = 53.50, *p* < 0.001; HC: 0.82 ± 0.42 vs. ID: 1.73 ± 0.71). Interestingly, both groups exhibited a perceptual underestimation of nocturnal awakenings, with subjectively reported densities consistently lower than those derived from PSG. The ID group, compared to HCs, also showed a greater density of cortical arousals (U = 121, *p* = 0.033; HC: 3.43 ± 2.00 vs. ID: 5.77 ± 4.15) (see Figure 1).

As expected, the TSTm index also significantly differed between groups, with ID participants displaying a more pronounced underestimation of their oTST (U = 305.00, *p* = 0.004; HC: 107 ± 29.49 vs. ID: 75.13 ± 30.9). Importantly, no HC met the TSTm threshold for significant misperception according to Lecci et al., 2020 [9], underscoring that pronounced SSM appears to be a phenomenon specific to ID in our sample. Moreover, oTST was significantly lower in IDs than in HCs (U = 307.00, *p* = 0.004; HC: 428.28 ± 83.41 vs. ID: 364.53 ± 72.85).

#### 3.1.3. Correlations Analysis

**Figure 2:**
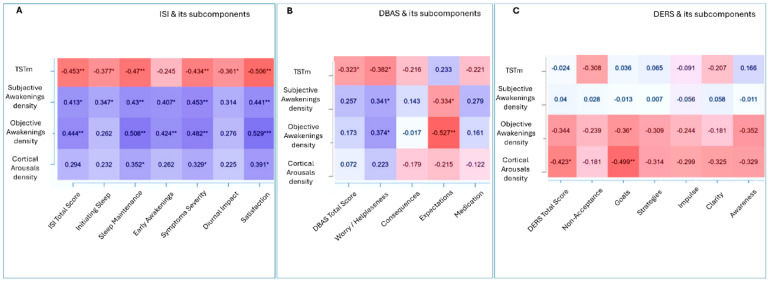
Heatmaps illustrating Spearman’s correlations between TSTm, nocturnal awakenings density (both subjective and objective), cortical arousal density, and psychological variables in both ID and HC groups: ISI and its subcomponents (**A**); DBAS and its subcomponents (**B**); DERS and its subcomponents (**C**). Positive associations are shown in shades of purple, with darker colors indicating stronger correlations. Negative associations are shown in shades of pink, with darker tones representing stronger inverse relationships. *p* < 0.05 *, *p* < 0.01 **, *p* < 0.001 ***. Abbreviations: dysfunctional beliefs about sleep (DBAS) and its subscales (consequences; worry/helplessness; expectations; medication); insomnia disorder (ID); DERS subscales: goals (Goals), strategies (Strategies), impulse (Impulse), non-acceptance of emotions (Non acceptance), emotional clarity (Clarity), emotional awareness (Awareness); insomnia severity index (ISI); healthy control (HC).

Exploratory correlation analyses reveal a coherent pattern linking TSTm and indices of sleep fragmentation to both subjective and objective sleep features, as well as psychological traits, across the entire sample, including both IDs and HCs. These correlations, presented as heatmaps (see Figure 2), are intended for descriptive purposes. For completeness, they are reported in Supplementary Results.

#### 3.1.4. Regression Analyses

To further elucidate the determinants of TSTm in ID, we conducted a stepwise multiple regression analysis with the TSTm index as the dependent variable. The independent variables included the total ISI score and its subcomponents, the total DERS score and its subcomponents, oAWKd, CAd, percentages of REM, N1 and N2 sleep, as well as objective sleep latency to N3 and REM stages. The final model accounted for 72.1% of the variance in TSTm (adjusted R^2^ = 0.721; RMSE = 11.793), indicating a robust explanatory capacity.

Greater underestimation of sleep (i.e., lower TSTm values) was significantly predicted by longer latency to N3 sleep (β = −0.531, *p* < 0.001), increased density of cortical arousals (β = −3.412, *p* = 0.002), and lower emotional clarity (DERS Clarity; β = −1.72, *p* = 0.018). These findings suggest that delayed initiation of deep sleep, subtle cortical arousal, and difficulties in identifying emotional states contribute to distorted sleep perception, leading to the subjective sense of prolonged wakefulness. In contrast, a higher proportion of REM sleep (β = +0.915, *p* = 0.016) and longer latency to REM sleep (β = +0.353, *p* = 0.001) were associated with higher TSTm scores, indicating more accurate or even overestimated sleep duration.

Taken together, the regression model reveals that greater underestimation of sleep (lower TSTm) is associated with delayed entry into deep sleep, increased cortical arousals, and lower emotional clarity, while a higher proportion of REM sleep and delayed REM onset appears to protect against misperception, possibly by supporting more stable or recognizable sleep architecture.

### 3.2. ID + TSTm vs. ID-TSTm

#### 3.2.1. Clinical and Psychological Variables

As part of our aim to explore potential differences between underestimators and normoestimators, we further analyzed the two ID subgroups: ID + TSTm (underestimators; n = 12, mean age = 46.83 ± 11.21; M:3/F:9) and ID-TSTm (normoestimators; n = 8, mean age = 38.50 ± 14.02; M:4/F:4). Importantly, this distinction was made solely based on the TSTm cut-off values defined in the Methods section [9], while oTST remained comparable between groups, underscoring that the subgrouping reflects differences in perception rather than in actual sleep duration.

**Figure 3:**
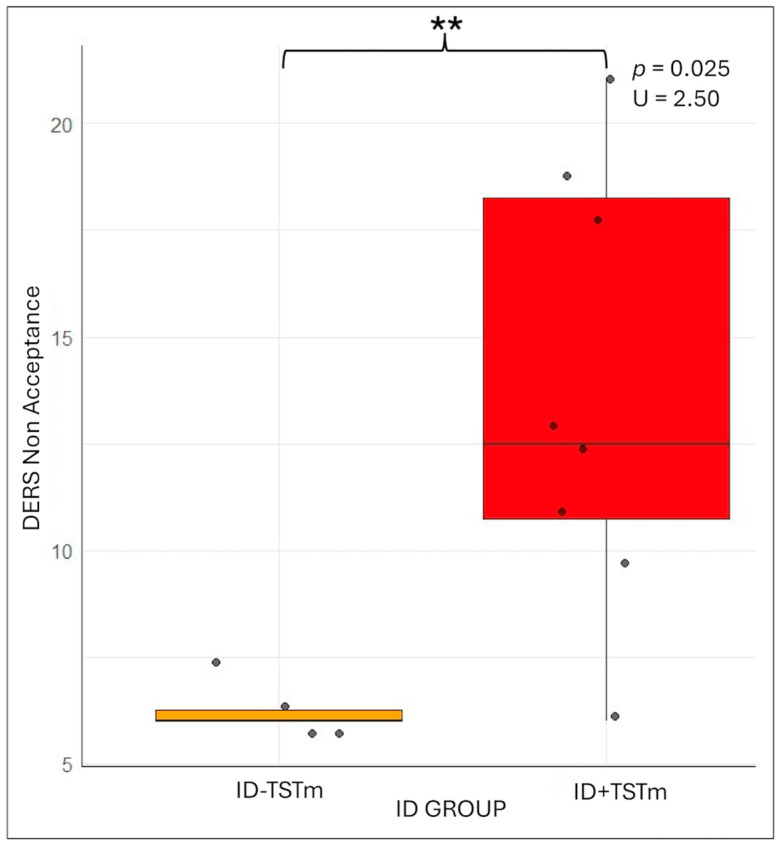
Results of the Mann–Whitney comparison between normoestimators (ID-TSTm in orange) and underestimators (ID + TSTm in red) on the DERS non-acceptance subscale. Abbreviations: subcomponent of difficulties in emotion regulation scale, non-acceptance (DERS Non-Acceptance); Insomnia Disorder (ID); ID normoestimators (ID-TSTm); ID underestimators (ID + TSTm). *p =* 0.001 **.

Mann–Whitney comparisons revealed a significant difference in the “non-acceptance” subscale of the DERS (U = 2.5, *p* = 0.025; means ± SD: ID-TSTm 6.25 ± 0.5 vs. ID + TSTm 13.75 ± 5.12), with higher scores for underestimators than normoestimators (see Figure 3). This variable did not emerge in the overall ID vs. HC analysis. While others psychological variables assessed through questionnaires were not found significant (*p* > 0.05).

#### 3.2.2. Subjective and Objective Sleep Variables

**Figure 4:**
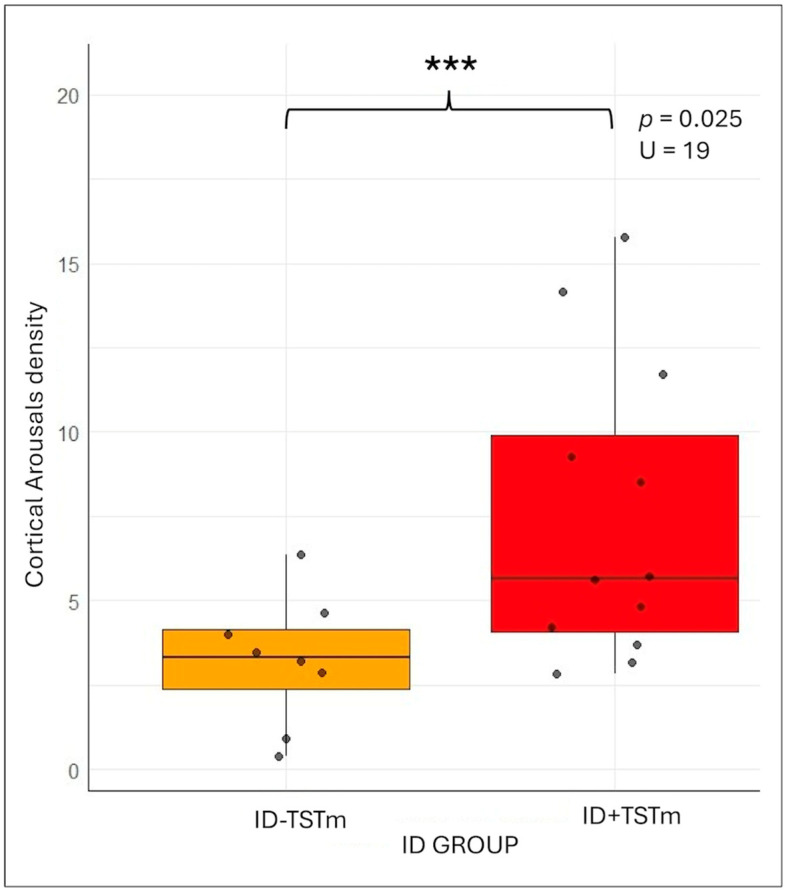
Results of the Mann–Whitney comparison on the number of cortical arousals between normoestimators (ID-TSTm in orange) and underestimators (ID-TSTm in red) within the insomnia disorder group. Abbreviations: Insomnia Disorder (ID); ID normoestimators (ID-TSTm); ID underestimators (ID + TSTm); density of cortical arousals (CAd). *p* < 0.001 ***.

Regarding sleep variables, subjectively, ID + TSTm participants reported significantly shorter sTST (U = 94.00, *p* < 0.001; ID + TSTm 387.5 ± 52.78 vs. ID-TSTm 201.67 ± 113.2) and poorer sSE (U = 90.5, *p* = 0.001; ID + TSTm 78.13 ± 10.82 vs. ID-TSTm 41.58 ± 23.17) during the experimental night. Consistently, sleep diaries reflected lower perceived sleep quality (U = 93, *p* < 0.001; ID + TSTm 6.63 ± 1.30 vs. ID-TSTm 2.42 ± 1.62) and greater perceived sleep disturbance (U = 8, *p* = 0.004; ID + TSTm 3.57 ± 2.15 vs. ID-TSTm 7.83 ± 2.52) in this subgroup. Objectively, underestimators spent more minutes in percentage in N1 sleep (U = 22, *p* = 0.047; ID + TSTm 7.12 ± 1.46 vs. ID-TSTm 11.33 ± 5.4), indicating differences in sleep continuity and depth that may relate to perceptual distortions. Interestingly, no significant difference emerged in the nocturnal awakening density (*p* > 0.05), both objective and subjective, despite its relevance in the broader comparison between IDs and HCs. Conversely, the cortical arousal density was significantly higher in ID + TSTm (U = 19.00, *p* < 0.025; ID + TSTm 3.23 ± 1.94 vs. ID-TSTm 7.46 ± 4.42), appearing as the only distinctive feature of the misperception profile and suggesting it may uniquely characterize this subgroup within the insomnia population (see Figure 4).

However, due to the limited sample size of the two subgroups, follow-up correlation and linear regression analyses were not conducted, as they would lack sufficient statistical power to yield reliable results.

## 4. Discussion

This study supports the conceptualization of SSM in ID as a phenomenon emerging from the interplay between subtle sleep fragmentation and emotional dysregulation. Our dimensional analyses suggest that TSTm varies along a continuum of physiological and cognitive vulnerability, whereas categorical comparisons highlight a more selective profile defined by CAd, N1%, and difficulties in emotional acceptance. Together, these findings challenge the notion of SSM as either a discrete clinical subtype or a mere proxy of insomnia severity, and instead support its interpretation as a partially dissociable vulnerability phenotype within ID.

Conventional macrostructural PSG metrics alone often fail to explain the perceptual distortions reported by patients. In current clinical practice, these indices may overlook key features of sleep instability that contribute to misperception.

Recent neurophysiological studies have linked SSM to cortical hyperarousal, reflected in increased high-frequency EEG activity, decreased delta power, and reduced delta/beta ratios, particularly during REM sleep [6,7,9]. Moreover, alterations in non-REM protective mechanisms, such as reduced amplitude and area of K-complexes, have also been associated with total sleep time underestimation, suggesting that impaired sleep protection may contribute to SSM [41].

However, such quantitative EEG indices require advanced signal processing, limiting their clinical applicability. We therefore focused on more accessible PSG-derived indicators of sleep instability, namely cortical arousal density and number of awakenings.

A key finding of our study is that oTST did not differ significantly between ID + TSTm and ID–TSTm, directly challenging the assumption that preserved sleep duration distinguishes underestimators from normoestimators. At the same time, both subgroups of ID showed shorter oTST compared to HCs. This result questions theoretical and methodological frameworks that define misperception as a discrepancy in sleep quantity alone, implicitly equating normal objective sleep with perceptual inaccuracy. Instead, it suggests that misperception can coexist with objectively shortened sleep, calling for a more nuanced interpretation of SSM in insomnia.

For example, Herzog et al., 2025 [42] classified underestimators through machine learning analysis of hypnodensities patterns. Although innovative, this model operationalizes SSM as the presence of insomnia complaints in the absence of objective sleep deficits, thereby reducing insomnia to a deviation from PSG-defined normality. Such an approach overlooks the diagnostic emphasis of the Diagnostic and Statistical Manual of Mental Disorders (5th ed.; DSM-5) [43] and ICSD-3 [2] on subjective complaints of poor or non-restorative sleep and their daytime consequences, regardless of objective sleep measures. Moreover, it overlooks the substantial night-to-night variability observed in ID, limiting the generalizability of one-night PSG-based classifications [44].

Similar issues arise in Fernandez-Mendoza et al., 2011 [21], which proposed a binary distinction between short objective sleep and misperceptive subtypes (i.e., normal objective sleep). This approach enforces a binary distinction that may wrongly pathologize normal variability in sleep perception. It also blurs the line between common under- or overestimation of sleep in the general population and clinically relevant misperception. Additionally, the reliance on fixed thresholds (e.g., <6 h of oTST) introduces arbitrary cut-offs and fails to capture the complexity of sleep instability in insomnia.

In contrast to categorical frameworks, Lecci et al., 2020 [9], proposed a more perceptually grounded and flexible model. Instead of applying external thresholds, they examined why some individuals consistently underestimate their sleep, regardless of clinical status. By linking the underestimation to EEG features— reduced delta and increased beta activity— they shift the focus from sleep quantity to the neurophysiological processes that shape subjective sleep experience. To capture this, they introduced the misperception index (i.e., TSTm), calculated as the ratio between subjective and objective TST, which offers a continuous, individualized measure of subjective-objective alignment.

We adopted this index because it anchors misperception in each individual’s own experience, rather than relying on priori thresholds or externally defined PSG norms. By capturing the subjective-objective alignment at the individual level, TSTm enables a person-centered evaluation of sleep misperception. This aligns with our broader conceptualization of SSM as a dynamic, affectively mediated phenomenon, shaped by sleep instability, emotional processing, and subjective evaluation. In this perspective, misperception is not an error but a meaningful, neurocognitively embedded feature of insomnia.

Among the PSG parameters that distinguished IDs from HCs, only cortical arousal density and N1 percentage also separated ID + TSTm from ID-TSTm. These findings align with prior evidence that frequent, subtle cortical arousals—especially in REM sleep—may disrupt the continuity of sleep experience without leading to full awakenings [9,45]. Such micro-events may amplify the salience of wake-like mentation, leading individuals to retrospectively interpret fragmented REM episodes as prolonged periods of wakefulness [14,46]. The higher N1 percentage in ID + TSTm further indicates lighter, less restorative sleep, which may hinder the consolidation of sleep as a cohesive and restful experience.

Interestingly, while the density of nocturnal awakenings (subjective and objective) distinguished IDs from HCs, it did not differ between ID + TSTm and ID-TSTm. This partially contrasts with findings by Castelnovo et al., 2021 [10], who observed more awakenings in high misperceivers. Our stricter thresholds for TSTm [9] may have produced clearer grouping and reduced overlap.

At the same time, our findings suggest that brief cortical arousals—although not meeting the threshold for full awakenings—may play a pivotal role in shaping the disrupted perception of sleep continuity among misperceivers. In our study, objective awakenings were defined as ≥60 s of wakefulness, implying that shorter arousals were not classified as awakenings, which excluded shorter arousals that may nonetheless have contributed to sleep fragmentation and the subjective sense of non-restorative sleep.

Psychologically, several self-report measures (e.g., DBAS, MCQI, PSS, BDI-II) distinguished IDs from HCs, yet emotional dysregulation did not emerge as a group-level marker of insomnia. At the subgroup level, however, a categorical comparison between underestimators (ID + TSTm) and normoestimators revealed greater difficulties in emotional acceptance among ID + TSTm. This DERS subscale captures the tendency to perceive negative emotions as intolerable or threatening, an attitude that may foster nocturnal hypervigilance toward internal cues and reinforce the perception of poor or insufficient sleep.

Increased cortical arousal density and difficulties in emotion regulation appear to interact synergistically in producing SSM. Emotion regulation difficulties—particularly poor emotional acceptance—amplify hyperarousal: individuals who perceive negative emotions as intolerable engage in nocturnal hypervigilance, which elevates cognitive arousal and in turn increases cortical arousal density [47,48,49,50]. This establishes a self-reinforcing loop: hyperarousal heightens emotional distress, while impaired regulation prevents its resolution, likely affecting retrospective evaluations of sleep continuity. Poor emotional clarity may worsen this cycle by limiting the capacity to place transient arousal (e.g., pre-sleep stress) in context, so that brief disturbances are misinterpreted as signs of prolonged wakefulness. Converging neuroimaging and EEG evidence further supports this model, showing that disrupted connectivity in limbic and salience networks, together with restless and fragmented REM sleep, undermines overnight emotional adaptation and contributes to persistent hyperarousal [47,48,50]. Recent cognitive-emotional models of insomnia explicitly conceptualize SSM as the outcome of this loop, where trait vulnerabilities (e.g., difficulties in emotion regulation) and state hyperarousal converge to sustain the subjective-objective sleep discrepancy [19,51].

Our stepwise regression model confirmed the dimensional convergence of physiological and psychological mechanisms in predicting the degree of SSM. Specifically, greater cortical arousal density, longer latency to N3, and reduced emotional clarity significantly predicted TSTm, together explaining over 70% of its variance. This highlights a robust, integrative profile wherein fragmented sleep and affective dysregulation jointly contribute to subjective-objective discrepancies in sleep.

However, categorical comparisons between ID + TSTm and ID–TSTm did not reveal significant differences in ISI scores. This discrepancy suggests that while the SSM is dimensionally associated with insomnia severity, individuals with pronounced misperception (ID + TSTm) do not necessarily exhibit a more severe clinical phenotype.

Rather, this subgroup may represent a distinct vulnerability profile, characterized by increased sensitivity to internal cues and emotional reactivity, rather than by the absolute intensity of insomnia symptoms. This interpretation aligns with the notion of SSM as an affectively loaded variant within the insomnia spectrum, rather than as a marker of symptom severity per se. Similarly, DBAS scores, especially the worry/helplessness dimension, correlated with TSTm, but did not show a significant difference categorically, indicating a gradient rather than a discrete pattern. It is important to note that our ID sample was characterized by generally high ISI scores, which may have limited the detection of subgroup differences (i.e., as due to a ceiling effect).

Moreover, metacognitive beliefs about sleep (MCQI), although significantly elevated in IDs compared to HCs, did not differ between ID + TSTm and ID-TSTm. This pattern reinforces the notion that while metacognitive vigilance may contribute to the development and persistence of insomnia, it is not a primary driver of misperception [16]. Instead, misperception appears more tightly coupled with state-level variations in emotional processing and arousal, rather than stable cognitive traits [52].

Several limitations warrant consideration. First, the relatively small sample size—particularly once IDs were further divided into underestimators and normoestimators— reduced statistical power and constrains the generalizability of our findings. While this approach is consistent with methodological recommendations for exploratory analyses in small samples [53,54], it increases the possibility of type I errors. Indeed, exploratory analyses are hypothesis-generating rather than confirmatory, and strict correction may obscure potentially interesting findings that warrant further investigation [55]. Accordingly, the associations we observed should be regarded as preliminary and ideally, they should be validated in subsequent confirmatory studies.

Third, sleep was assessed using a single-night PSG. Although single-night PSG can capture misperception of total sleep time at the group level [18], evidence indicates that SSM shows night-to-night variability and environmental sensitivity [25,56]. These factors underscore the exploratory nature of our study and the need for replication in larger, multi-night investigations. Future studies should also incorporate nocturnal mentation assessments, including dream recall, phenomenology, and emotional valence, to examine whether SSM is associated with a specific profile of sleep-related consciousness. Prior work suggests that misperceivers may show reduced dream recall or predominantly thought-like mentation [57], making this a promising avenue for further research.

## 5. Conclusions

Taken together, our findings and the recent literature call for a critical reconsideration of how the divergence between subjective and objective sleep is defined and framed. The commonly used term sleep state misperception implies a misjudgment or error on the part of the individual, potentially invalidating the subjective experience of poor sleep. This framing neglects not only the complexity and legitimacy of subjective sleep complaints, but also the limitations of conventional objective measures, particularly standard PSG parameters, which may fail to capture subtle neurophysiological alterations underlying the subjective sense of wakefulness. In light of this, and building on the notion of mismeasurement [11], we support the shift proposed by Bensen-Boakes et al., 2022 [58], who advocate replacing sleep state misperception with the more neutral term sleep–wake state discrepancy. This alternative terminology better reflects the multidimensional nature of the subjective–objective gap, avoids pathologizing the patient’s perspective, and promotes a more respectful and accurate clinical and scientific discourse.

In sum, this study advances a dimensional and integrative model of SSM, highlighting its roots in sleep instability and emotional dysregulation, rather than in global reductions in sleep duration. Our findings challenge duration-based definitions of misperception and underscore the importance of markers such as cortical arousals, shallow N1 sleep, and emotion regulation difficulties, particularly non-acceptance and lack of clarity.

Rather than a mere perceptual error, SSM emerges as an expression of heightened emotional and physiological vulnerability within insomnia. This perspective opens interesting clinical avenues: instead of focusing solely on sleep, interventions might benefit from targeting affective reactivity and arousal sensitivity. Emotion-focused and acceptance-based approaches, in particular, warrant further investigation as potentially valuable strategies for this subgroup.

## Figures and Tables

**Table 1:** Psychological assessments included in the study.

Psychological Test	Abbreviation	Definition
Insomnia Severity Index [24]	ISI	Three core items assessing specific aspects of insomnia symptoms were analyzed: difficulty initiating sleep (item 1-a), difficulty maintaining sleep (item 1-b), and early morning awakenings (item 1-c). Together, these items constitute the Symptom Severity subscale. Two additional dimensions were also considered: Satisfaction, which evaluates the individual’s dissatisfaction with their current sleep pattern, and Diurnal Impact, which measures the perceived consequences of sleep difficulties on daytime functioning and their visibility to others.
Dysfunctional Beliefs and Attitudes about Sleep [31]	DBAS	It assesses maladaptive beliefs and attitudes related to sleep, reflecting (a) misconceptions or amplifications about the causes and the consequences of insomnia (Consequences); (b) unrealistic sleep expectations (Expectations); (c) diminished perceptions of control and predictability of sleep (Worry/Helplessness); and (d) faulty beliefs about sleep-promoting practices (Medication) [32].
Perceived Stress Scale [33]	PSS	It measures the extent to which individuals perceive their lives as stressful.
Beck Depression Inventory-II [34]	BDI-II	It assesses the severity of depressive symptoms.
State-Trait Anxiety Inventory [35]	STAY-I	It differentiates between temporary (state) and enduring (trait) anxiety.
the Difficulties in Emotion Regulation Scale—36 items [22]	DERS-36	It evaluates various aspects of emotional dysregulation across six subcomponents: (a) awareness and (b) understanding of emotions (Awareness; Clarity); (b) non acceptance of emotions (Non acceptance); (c) the ability to engage in goal-directed behavior (Goals), and refrain from impulsive behavior, when experiencing negative emotions (Impulse); and (d) access to emotion regulation strategies perceived as effective (Strategies) [36].
Metacognitions Questionnaire Insomnia [23]	MCQI	It assesses the metacognitive beliefs and processes associated with insomnia.

**Table 2:** Objective sleep parameters derived from PSG, with abbreviations and units of measurement.

Parameter	Abbreviation	Unit
Sleep Latency to N2, N3, REM	oSL	Minutes
Total Sleep Time	oTST	Minutes
Sleep Stage Percentages	N1%, N2%, N3%, REM%, NREM%	Percentage (%)
Wake After Sleep Onset	WASO	Minutes
Nocturnal Awakenings	oAWK	Count
Cortical Arousals	CA	Count
Time In Bed	TIB	Minutes
Sleep Efficiency (oTST/TIB) × 100	SE	Percentage (%)
Awakening Density oAWK is divided by total hours of sleep.	oAWK_d	Events/hour
Arousal Density CA is divided by total hours of sleep.	CA_d	Events/hour

**Table 3:** Subjective sleep parameters obtained from sleep diaries with their abbreviations and unit of measurement.

Parameter	Abbreviation	Unit
Subjective Sleep Latency	sSL	Minutes
Subjective Total Sleep Time	sTST	Minutes
Subjective Nocturnal Awakenings	sAWK	Count
Awakening Density (Subjective) sAWK is divided by total hours of self-reported sleep.	sAWK_d	Events/hour
Subjective Wake After Sleep Onset	sWASO	Minutes
Subjective Time in Bed	sTIB	Hours
Subjective Sleep Efficiency (sTST/sTIB) × 100	sSE	Percentage (%)
Sleep Disturbance	—	Likert (0–10)
Sleep Quality	—	Likert (0–10)

**Table 4:** Comparison of psychological and sleep-related questionnaire scores between individuals with ID and HC.

	HC Means ± SD (*N* = 20)	ID Means ± SD	U-Test	*p*-Value
Gender	10 M/10 F	7 M/13 F	170	0.352
Age	41.05 ± 11.55 (26–63)	43.50 ± 12.75 (18–65)	174	0.490
ESS	5.55 ± 2.95	4.44 ± 4.96	241.000	0.076
ISI	4.25 ± 2.38	16.94 ± 4.51	0.000	**<0.001**
ISI-1a	0.35 ± 0.59	1.53 ± 1.23	75.500	**0.002**
ISI-1b	0.3 ± 0.47	2.88 ± 0.93	3.000	**<0.001**
ISI-1c	0.5 ± 0.61	2.77 ± 1.09	16.500	**<0.001**
ISI_Symptoms Severity	1.15 ± 1.14	7.18 ± 2.07	1.500	**<0.001**
ISI_Diurnal Impact	1.35 ± 1.42	6.47 ± 3	21.500	**<0.001**
ISI_Satisfaction	2.1 ± 1.33	7.28 ± 2.27	3.000	**<0.001**
DBAS	51.3 ± 16.66	80.11 ± 23.82	56.500	**<0.001**
DBAS_Consequences	16.75 ± 7.43	24.13 ± 12.88	93.000	**0.034**
DBAS_Worry/Helplessness	14.3 ± 9.63	33-38 ± 9.23	26.500	**<0.001**
DBAS_Expectations	13.75 ± 3.8	9.1 ± 5.7	238.000	**0.013**
DBAS_Medication	6.5 ± 3.44	11.38 ± 5.6	70.000	**0.004**
BDI-II	6.75 ± 5.6	12.47 ± 8.57	98.500	**0.030**
PSS	14.35 ± 4.84	19.13 ± 5.73	81.000	**0.012**
DERS	72.85 ± 20.85	72.42 ± 20.9	121.000	0.984
DERS_Non-acceptance	11.2 ± 4.87	11.25 ± 5.51	127.500	0.784
DERS_Goals	11.05 ± 3.46	11.25 ± 3.57	124.000	0.891
DERS_Strategies	14.1 ± 5.1	13.83 ± 4.84	124.000	0.891
DERS_Impulse	10.3 ± 3.8	10.83 ± 3.76	105.000	0.570
DERS_Clarity	9.85 ± 3.42	10.33 ± 3.7	108.500	0.666
DERS_Awarness	8.25 ± 4.71	6.67 ± 3.11	144.000	0.357
TOT STAY-1s	36.05 ± 8.4	42.47 ±10.71	108.500	0.063
TOT STAY-2t	42.8 ± 4.99	46.24 ± 9.1	121.500	0.142
MCQ-I	96.79 ± 22.73	123.27 ± 28.94	79.000	**0.029**

Significant main effects are in bold. For the ID group, sample size (n) varies slightly across measures due to missing data, see Methods Section (“Missing Data”) for full details. Abbreviations: Beck Depression Inventory (BDI); Dysfunctional Beliefs and Attitudes about Sleep (DBAS); Difficulties in Emotion Regulation Scale (DERS); Healthy Controls (HC); Insomnia Disorder (ID); Insomnia Severity Index (ISI); Metacognitions Questionnaire—Insomnia (MCQI); Pittsburgh Sleep Quality Index (PSQI); Perceived Stress Scale (PSS).

## Data Availability

The conditions of our ethics approval do not permit public archiving of the anonymized study data. Readers seeking access to the data should contact the corresponding author. Access will be granted to named individuals in accordance with ethical procedures governing the reuse of sensitive data. Specifically, requestors must complete a formal data-sharing agreement.

## Supplementary Materials

The following supporting information can be downloaded at: https://www.mdpi.com/article/10.3390/brainsci15101078/s1.
