# Supplementary material for: Sleep State Misperception in Insomnia: The Role of Sleep Instability and Emotional Dysregulation
Source: Brain Sci. 2025 Oct 4;15(10):1078. doi: 10.3390/brainsci15101078 (PMC12563266; doi:10.3390/brainsci15101078)
Supplement: Supplementary file 1 [file brainsci-15-01078-s001.zip › brainsci-3855870-supplementary.pdf]

brain sciences

MDPI

Supplementary Materials

Sleep State Misperception in Insomnia: The Role of Sleep Instability and Emotional Dysregulation

Elettra Cini [1,2], Francesca Bolengo [1,2], Elisabetta Fasiello [3], Francesca Berra [1,2], Maurizio Gorgoni [4,5], Marco Sforza [2], Francesca Casoni [2], Paola Proserpio [2], Vincenza Castronovo [2], Luigi De Gennaro [4,5], Luigi Ferini-Strambi [1,2] and Andrea Galbiati [1,2*]

[1] Faculty of Psychology, Vita-Salute San Raffaele University, Milan, Italy elettra.cini@gmail.com (E.C.); francibolengo@gmail.com (F.B.); francescaberra1997@gmail.com (F.B.); ferinistrambi.luigi@hsr.it (L.F.-S.)
[2] Department of Clinical Neurosciences, Neurology-Sleep Disorders Center, IRCCS San Raffaele Scientific Institute, Milan, Italy; marco.sforza@gmail.com (M.S.); casoni.francesca@hsr.it (F.C.); proserpio.paola@hsr.it (P.P.); castronovo.vincenza@hsr.it (V.C.)
[3] IUSS Cognitive Neuroscience (ICON) Center, Scuola Universitaria Superiore IUSS, Pavia, Italy elisabettafasiello91@gmail.com (E.F.)
[4] Department of Psychology, Sapienza University of Rome, Rome, Italy; luigi.degennaro@uniroma1.it (L.D.-G.); maurizio.gorgoni@uniroma1.it (M.G.)
[5] Body and Action Lab, IRCCS Fondazione Santa Lucia, Rome, Italy
* Correspondence: andrea.galbiati.unisr@gmail.com; Tel.: +39-022-643-3397

1. Supplementary Results

Exploratory correlation analyses were conducted for descriptive purposes. Heatmaps of the correlations (Figure S1) are reported. These analyses were not included in the main text, as regression models were considered more appropriate to address the study hypotheses.

Correlation analyses revealed a coherent pattern linking TSTm and indices of sleep fragmentation to both subjective and objective sleep features, as well as psychological traits, across the entire sample, including both IDs and HCs (see Fig 2).

From a subjective perspective, more accurate sleep perception (i.e., higher TSTm values) was positively correlated with shorter sSL ($\varrho = 0.347$, $p = 0.028$), lower sWASO ($\varrho = 0.479$, $p = 0.002$), and higher sSE ($\varrho = 0.682$, $p <.001$).

Consistently, objective PSG parameters revealed that more accurate sleep estimation was negatively associated with longer latency to N3 stage ($\varrho = -0.349$, $p = 0.027$), as well as decreased proportions of lighter sleep stages such as N1 ($\varrho = -0.321$, $p = 0.044$), N2 ($\varrho = -0.319$, $p = 0.045$), and overall NREM sleep ($\varrho = -0.354$, $p = 0.027$). Moreover, a better accuracy of sleep estimation was positively related to higher percentages of REM sleep ($\varrho = 0.354$, $p = 0.027$) and better oSE ($\varrho = 0.345$, $p = 0.029$).

Regarding sleep fragmentation, better accuracy in sleep perception negatively correlated with cortical arousal density ($\varrho = -0.311$, $p = 0.05$) and with objective nocturnal awakenings density (oAWKd; $\varrho = -0.362$, $p = 0.023$). While no significant correlation emerged between TSTm and subjective nocturnal awakenings density, sAWKd positively correlated with oAWKd ($\varrho = 0.587$, $p < .001$). All three fragmentation markers (CAd, oAWKd, sAWKd) were positively associated with N1% ($\varrho = 0.563$, $p <.001$; $\varrho = 0.709$, $p <.001$; $\varrho = 0.363$, $p = 0.021$, respectively) and only CAd and oAWKd positively correlated with objective latency to N3 stage (CAd, $\varrho = 0.625$, $p <.001$; oAWKd, $\varrho = 0.358$, $p = 0.023$),

indicating that greater arousal and awakening densities are both linked to lighter sleep and delayed onset of deep sleep. Moreover, both oAWKd and sAWKd were negatively related to reduced SE% (ϱ = –0.644, p < .001; ϱ = –0.409, p = 0.009) and positively related to oWASO (ϱ = 0.595, p < .001; ϱ = 0.393, p = 0.013).

On the psychological side, better accuracy in sleep perception was negatively associated with higher insomnia severity, as captured by ISI total score (ϱ = –0.453, *p* = 0.005), and specific components including difficulties to fall asleep (ISI-1a; ϱ = –0.377, *p* = 0.022), sleep maintenance (ISI-1b; ϱ = –0.47, *p* = 0.003), symptom severity (ϱ = –0.434, *p* = 0.007), diurnal impairment (ϱ = –0.361, *p* = 0.028), and sleep dissatisfaction (ϱ = –0.506, *p* = 0.001). DBAS also showed significant negative correlations with accuracy in sleep perception, both for total score (ϱ = –0.323, *p* = 0.048) and the worry/helplessness subcomponent (ϱ = –0.382, *p* = 0.021).

Fragmentation indices, particularly oAWKd and sAWKd, were also significantly associated with psychological distress. They correlated positively with ISI total score (ϱ = 0.444, *p* = 0.007; ϱ = 0.413, *p* = 0.011, respectively), DBAS worry/helplessness (ϱ = 0.374, *p* = 0.027; ϱ = 0.341, *p* = 0.042), and negatively with DBAS expectations (ϱ = –0.527, *p* = 0.001; ϱ = –0.341, *p* = 0.047).

CAd also showed significant positive correlations with sleep maintenance (ISI-1b; ϱ = 0.352, *p* = 0.033) and ISI satisfaction (ϱ = 0.391, *p* = 0.015), and was negatively correlated with worse emotional regulation, including total DERS score (ϱ = –0.423, *p* = 0.016) and the DERS goals subscale (ϱ = –0.499, *p* = 0.004). Similarly, DERS-goals also negatively correlated with oAWKd (ϱ = –0.36, *p* = 0.047).
